# Cross-section perimeter is a suitable parameter to describe the effects of different baffle geometries in shaken microtiter plates

**DOI:** 10.1186/1754-1611-8-18

**Published:** 2014-07-15

**Authors:** Clemens Lattermann, Matthias Funke, Sven Hansen, Sylvia Diederichs, Jochen Büchs

**Affiliations:** 1 AVT.Biochemical Engineering, RWTH Aachen University, Worringer Weg 1, 52074 Aachen, Germany; 2 Lonza Group Ltd, Münchensteinerstraße 38, 4002 Basel, Switzerland; 3 Evonik Industries AG, Rodenbacher Chaussee 4, 63457 Hanau-Wolfgang, Germany

**Keywords:** Shaken bioreactors, Maximum oxygen transfer capacity (OTR_max_), Degree of baffling, Relative perimeter, Out-of-phase phenomena

## Abstract

**Background:**

Biotechnological screening processes are performed since more than 8 decades in small scale shaken bioreactors like shake flasks or microtiter plates. One of the major issues of such reactors is the sufficient oxygen supply of suspended microorganisms. Oxygen transfer into the bulk liquid can in general be increased by introducing suitable baffles at the reactor wall. However, a comprehensive and systematic characterization of baffled shaken bioreactors has never been carried out so far. Baffles often differ in number, size and shape. The exact geometry of baffles in glass lab ware like shake flasks is very difficult to reproduce from piece to piece due to the hard to control flow behavior of molten glass during manufacturing. Thus, reproducibility of the maximum oxygen transfer capacity in such baffled shake flasks is hardly given.

**Results:**

As a first step to systematically elucidate the general effect of different baffle geometries on shaken bioreactor performance, the maximum oxygen transfer capacity (OTR_max_) in baffled 48-well microtiter plates as shaken model reactor was characterized. This type of bioreactor made of plastic material was chosen, as the exact geometry of the baffles can be fabricated by highly reproducible laser cutting. As a result, thirty different geometries were investigated regarding their maximum oxygen transfer capacity (OTR_max_) and liquid distribution during shaking. The relative perimeter of the cross-section area as new fundamental geometric key parameter is introduced. An empirical correlation for the OTR_max_ as function of the relative perimeter, shaking frequency and filling volume is derived. For the first time, this correlation allows a systematic description of the maximum oxygen transfer capacity in baffled microtiter plates.

**Conclusions:**

Calculated and experimentally determined OTR_max_ values agree within ± 30% accuracy. Furthermore, undesired out-of-phase operating conditions can be identified by using the relative perimeter as key parameter. Finally, an optimum well geometry characterized by an increased perimeter of 10% compared to the unbaffled round geometry is identified. This study may also assist to comprehensively describe and optimize the baffles of shake flasks in future.

## Introduction

Shaken small scale bioreactors like shake flasks or microtiter plates are typically used for high throughput screening processes today. In the last years, many efforts have been made to characterize small scale bioreactors by describing and modeling liquid distribution, gas transfer, specific power input as well as mixing (reviewed in [1-7]). These efforts are motivated by the need to understand the yet insufficiently characterized screening systems in more detail. Furthermore, knowledge about important process parameters in an earlier stage of process development is desired [7,8]. While shake flasks are already characterized to some extent, there are several open questions regarding microtiter plates. The small dimensions of these reactor systems result in specific problems like the influence of surface tension or the lack of space for on-line measurement equipment.

For successful screening processes, sufficient oxygen supply is mandatory [7]. In shaken bioreactors higher maximum oxygen transfer capacities can be achieved by reducing the filling volume or increasing the shaking frequency, the vessel diameter or the shaking diameter. However, this approach has clear limits. Another possibility to enhance the maximum oxygen transfer capacity is the use of baffles inside the bioreactor. However, for shake flasks it is known that the reproducibility of oxygen transfer is poor between individual flasks if baffles are introduced [7,9,10]. This is due to the flow characteristics of molten pyrex glass which is very hard to control during the fabrication process of the baffles. As a result, it is nearly impossible to reproduce the exact geometries of the baffles. In most cases this can be proven even by the naked eye. Therefore, some authors even do not recommend the use of baffled shaken bioreactors at all [7,11].

Fabrication of baffles in microtiter plates is much more accurate and reproducible if laser cutting or injection molding is used. A wide variety of different baffled microtiter plates were extensively studied by Funke et al. [12]. An optimal geometry was found, considering different criteria. The best version is meanwhile commercially available. It shows reproducible oxygen transfer properties on an elevated level.

Up to now, no general method to predict the impact of baffles and its geometries on the maximum oxygen transfer capacity of shaken bioreactors is available. This is mainly caused by the problem to obtain a set of different baffled bioreactors with reproducible geometries made from glass. Therefore, in this work we utilized the results of Funke et al. [12] which were generated with the sulfite method for a wide variety of different reproducible geometries of 48-well microtiter plates as shaken model bioreactors. By using 48-well microtiter plates, an influence of surface tension which is quite prominent in 96-well microtiter plates is minimized [4,13]. The absolute cross-section areas were consistently kept constant (112 mm^2^) among the different geometries. The relationship between the geometries of the wells and the maximum oxygen transfer capacities were derived and a respective mathematical correlation was developed. This correlation depends on the shaking frequency, the filling volume and the relative perimeter of the cross-section area of the wells as new geometric key parameter. In this equation, the relative perimeter is the only criterion reflecting the degree of baffling. Thereby, all important parameters of baffling like number, size and shape of the baffles are considered in one value. This work is deemed to be the first step in understanding and quantifying the impact of baffles on shaken bioreactor performance. In a future attempt the applicability of the developed approach has to be proven also for baffled shake flasks.

## Results and discussion

### Influence of baffling on oxygen transfer in microtiter plates

**Figure 1:**
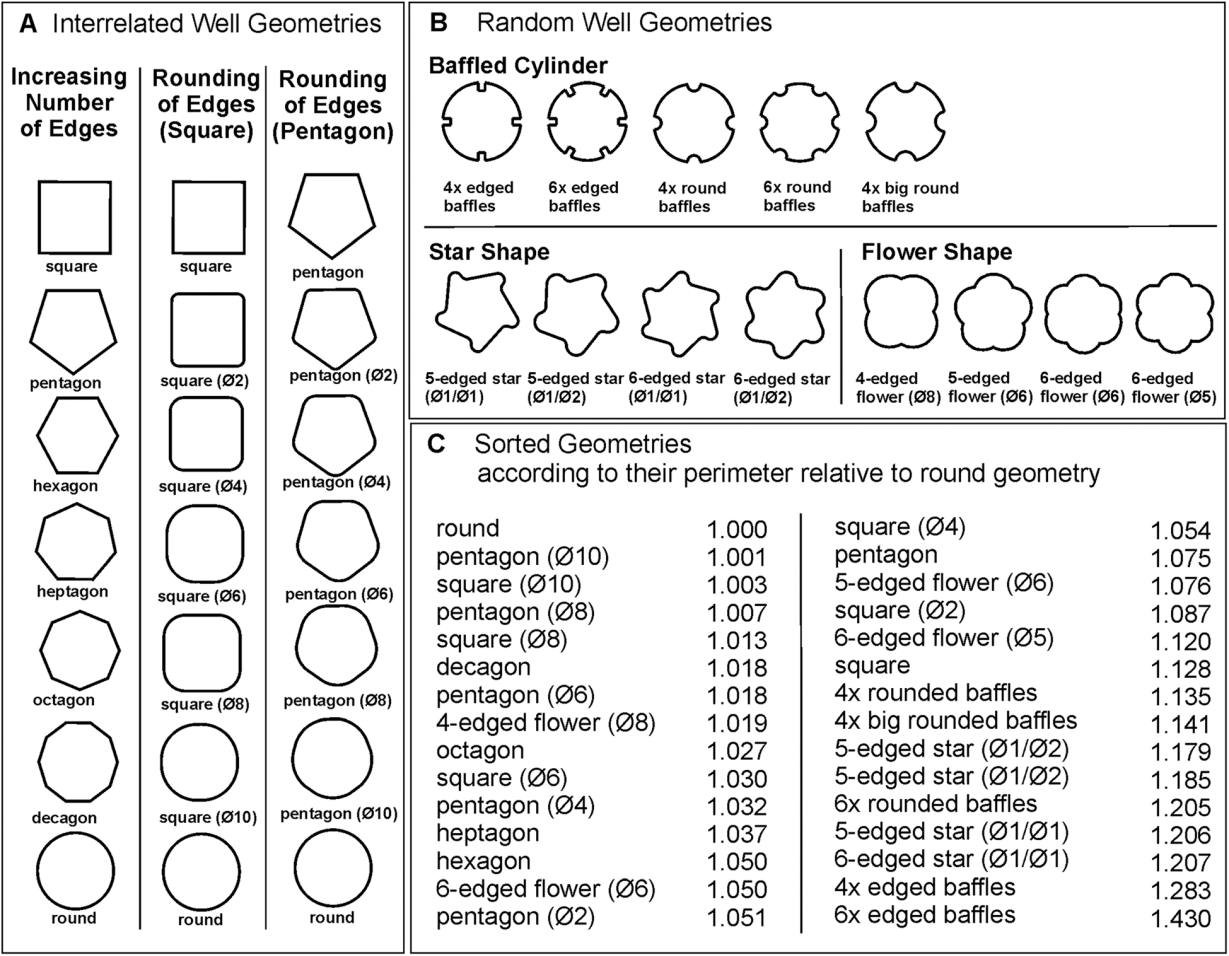
**Designs of MTP well geometries (48-well plate format). (A)** 3 different sets of well geometries represent the gradual transition from the most pronounced baffling (square and pentagon geometry, respectively) to the least pronounced geometry (round geometry); **(B)** 3 groups of well designs attained by introducing different types of baffles in round (baffled cylinders) or edged (star and flower shapes) well geometries. All geometries have an equivalent cross-section area of 112 mm^2^. The diameters of the edge roundings or the introduced half-circular baffles are given in parentheses. Further design details can be found in Funke et al. (2009) [12]; **(C)** Geometries described in (A) and (B) sorted according to their perimeter relative to the round well geometry.

**Figure 2:**
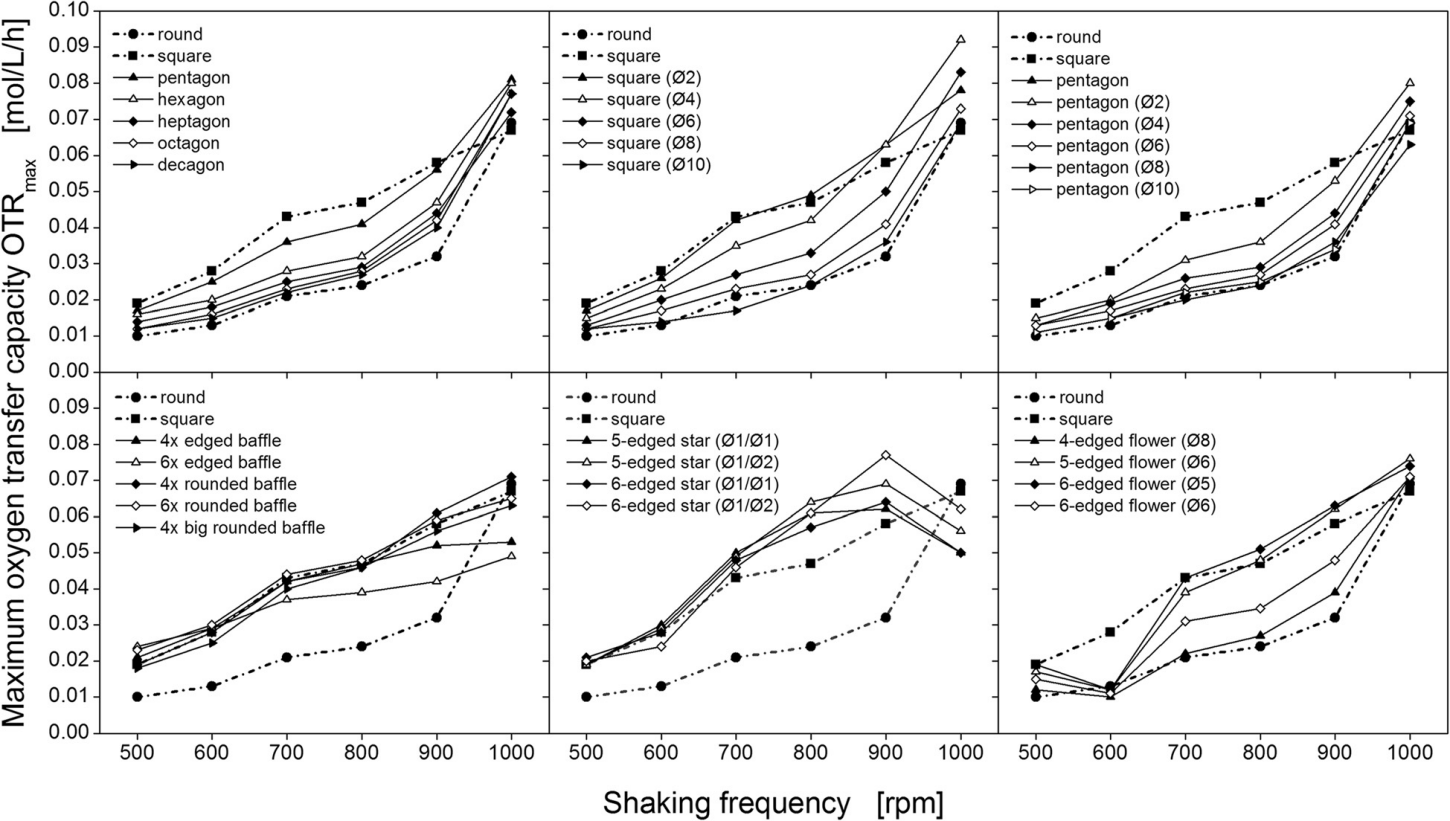
**Maximum oxygen transfer capacity (OTR**_**max**_**) obtained by the sulfite system at increasing shaking frequencies.** OTR_max_ values are determined for different well geometries with 112 mm^2^ cross-section area (48-well plate format). The filling volume is kept constant at 600 μL. Measurements performed with a modified BioLector measurement system: shaking diameter: 3 mm, temperature: 25°C, shaking frequency: 500 – 1000 rpm.

In this study, the maximum oxygen transfer capacity OTR_max_ of thirty different cross-section geometries in 48-well microtiter plates varying in their degree of baffling, as shown in Figure 1, were investigated. In Figure 2 the OTR_max_ values of all geometries are exemplarily shown for a filling volume of 600 μL at increasing shaking frequencies. The OTR_max_ increases with increasing shaking frequency. Furthermore, a clear dependency between the maximum oxygen transfer capacity and the cross-section geometry of the wells can be noticed. In particular, the OTR_max_ values of the baffled geometries shown in Figure 1A (transition edged to round) increases between least pronounced baffling (round) and most pronounced baffling (square) for nearly all shaking frequencies (compare upper diagrams in Figure 2). It can be concluded, that the OTR_max_ systematically depends on the applied baffle geometry. However, until now only an empirical description of the performance of the different baffle designs implemented in this study was available [12]. A mathematical correlation between the degree of baffling and the OTR_max_ could not yet be established. Moreover, the unexpected stagnation or decrease of the OTR_max_ with increasing shaking frequency, as it can be observed for some well geometry in Figure 2 (compare edged star geometry, middle of bottom diagram), could not be explained.

The influence of baffling on the maximum oxygen transfer capacity can be described, if a geometric key parameter is found, which allows an overall description of different cross-section geometries. For the well designs resulting from the transition from edged to round geometry (Figure 1A) an adequate parameter could be the number of edges or the diameter of the edge roundings. With both parameters, however, it is not possible to adequately correlate all cross-section geometries and measured maximum oxygen transfer capacities (data not shown). The description of the other well geometries shown in Figure 1B is even more difficult with this approach. A more universal key parameter is required. Therefore, various further parameters like the ratio between maximum and minimal diameter have been investigated (data not shown). Finally, the perimeter of the cross-section area of the wells has been identified as the parameter resulting in the best correlation between different cross-section geometries and measured OTR_max_ values in the investigated microtiter plates. For comparison, we normalized the perimeter of the baffled geometry through dividing by the perimeter of the round reference geometry:

(1) $$\text{Peri}:=\;\frac{\text{Perimete}r_{\text{baffled}}}{\text{Perimete}r_{\text{round}}}.$$

**Figure 3:**
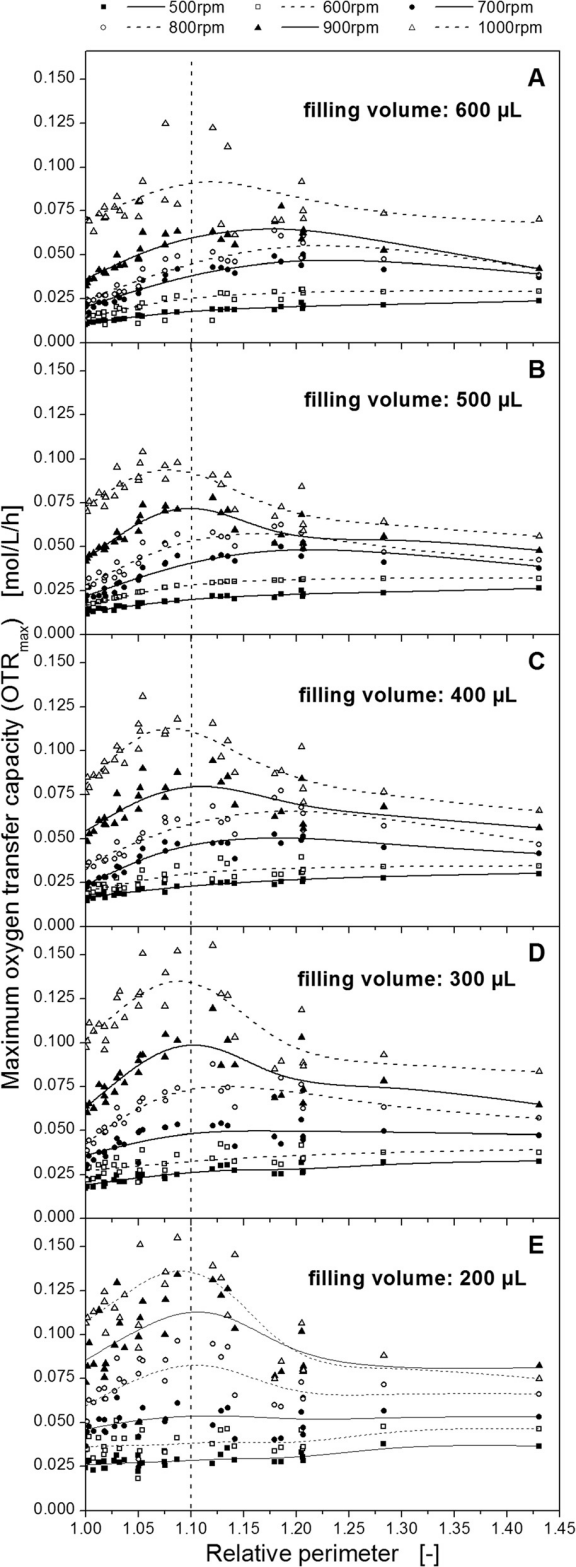
**Maximum oxygen transfer capacity (OTR**_**max**_**) as a function of the relative perimeter.** The perimeters of the different well geometries are related to the perimeter of the round geometry with the same well cross-section area of 112 mm^2^. The dashed line represents the transition from in-phase to out-of-phase operation condition. Measurements performed with a modified BioLector measurement system: shaking diameter: 3 mm, temperature: 25°C, shaking frequency: 500 – 1000 rpm, filling volume: 600 μL **(A)**, 500 μL **(B)**, 400 μL **(C)**, 300 μL **(D)**, 200 μL **(E)**.

Eq. (1) represents a universal, dimensionless parameter which includes the number as well as the size and the shape of the baffles. For example, the relative perimeter of highly pronounced baffles with a steep slope is greater than that of less pronounced baffles. Also, the number of baffles is considered by using the relative perimeter as correlating parameter. This property characterizes the relative perimeter as a powerful parameter allowing a ranking of investigated geometries as shown in Figure 1C. Furthermore, the relative perimeter enables the pooling of all measurement data for one filling volume (as shown in Figure 2) in one diagram (Figure 3). Thus, the maximum oxygen transfer capacity for all different geometries can easily be compared.

In Figure 3, the OTR_max_ is plotted as a function of the relative perimeter of all investigated geometries to systematically characterize the influence of baffles on the maximum oxygen transfer capacity. The smoothing splines between the measurement points were calculated by applying a modified L-curve criterion. As expected, the maximum oxygen transfer capacity increases with decreasing filling volume, as illustrated in Figure 3. This result agrees with the theory and literature, since the surface-to-volume ratio, and, thus, the oxygen transfer into the bulk liquid increases with decreasing filling volume in surface aerated bioreactors [11]. The measured OTR_max_ value in general increases with increasing shaking frequency. This trend also agrees with literature [14]. Furthermore, the shapes of the OTR_max_ curves over the relative perimeter for filling volumes of 200 - 400 μL equal each other. For example, looking at a specific shaking frequency, the OTR_max_ curves are merely shifted in their absolute height depending on the filling volume. For filling volumes of 500 μL and 600 μL the shape of the OTR_max_ curves seem to be smoother.

The most important result depicted in Figure 3 is the development of the OTR_max_ curves as a function of the relative perimeter. Starting at a relative perimeter of 1.0, up to a relative perimeter of approximately 1.05, a linear increase of the OTR_max_ values can generally be observed. Subsequently, always a maximum is reached at a relative perimeter of about 1.075 to 1.12. For even higher relative perimeters, the OTR_max_ values remain either constant (<700 rpm) or decrease (>700 rpm). Obviously, there is a common limit of the OTR_max_ for all applied shaking frequencies, filling volumes and well geometries. Expressed in other words, increasing the degree of baffling increases the maximum oxygen transfer capacity until an upper limit value is reached. This maximum is generally observed at baffled geometries with a perimeter 10% larger than the one of the unbaffled round geometry. The result is quite astonishing because it reflects the abstract character of the relative perimeter, taking number, size and shape of the baffles indirectly into account. According to Figure 1C, the maximum oxygen transfer capacity at a relative perimeter of 1.1 corresponds to a geometry between square (Ø2) and the 6-edged flower (Ø5). It is remarkable that this result excellently agrees with the qualitative observations made by Funke et al [12]. It can now be quantitatively described by means of the relative perimeter.

### Influence of baffling on liquid distribution in microtiter plates

The phenomenon of stagnant or decreasing OTR_max_ values at relative perimeters > 1.1 in Figure 3 has to be evaluated in detail to entirely explain the whole data. In order to understand the mass transfer data, the behaviour of the rotating liquid has to be considered. The bulk liquid in shaken bioreactors typically rotates inside the vessel if in-phase conditions exist. In contrast, at out-of-phase conditions the major part of the liquid remains on the bottom of the vessel and does not move anymore. Simultaneously, the maximum liquid height and the maximum oxygen transfer capacity into the liquid is reduced.

Three different reasons for inducing these unsuitable out-of-phase conditions in shake flasks were identified in literature [15]. One effector is elevated viscosity. In this case, out-of-phase conditions are provoked, if the rotating centrifugal force is not strong enough to overcome the viscous forces [16]. A new Phase-Number Ph was defined and concluded that Ph > 1.26 is the relevant constraint for desired in-phase operation [16]. Another reason for out-of-phase conditions are unsuitable high ratios between the maximum diameter of the shaken bioreactors and the shaking diameter of the applied shaker, as shown for non-baffled large shake flasks by Büchs et al. [15] and for microtiter plate with round geometry by Kensy et al. [17]. The third reason for unsuitable out-of-phase operating conditions in shake flasks is the introduction of baffles which are too in number large in size. Problems may already occure at water-like viscosities. The probability of out-of-phase conditions increases with decreasing shaking diameter [7,15]. The undesired phenomenon appears if the rotating centrifugal force generated by the shaker is not strong enough to overcome the negative impact of the baffles preventing the bulk liquid from rotating in the flask. Although this third reason for out-of-phase conditions resembles the first reason, the above mentioned Phase-Number can not be used to evaluate the impact of baffles.

Considering the decreasing mass transfer at higher degrees of baffling (relative perimeter > 1.1) it can be speculated that out-of-phase conditions exist at relative perimeters higher than 1.1 in the investigated 48-well microtiter plates. Therefore, an experimental examination of the appearance of out-of-phase conditions has been conducted in this study. As mentioned above, the shaken liquid remains at the bottom of the well if out-of-phase conditions are present. This implies that the liquid height at the well center is elevated compared to in-phase conditions. Hence, an optical measurement of the liquid height provides further information about the flow conditions in the microtiter wells and, thus, may help to explain the observations of the mass transfer study.

**Figure 4:**
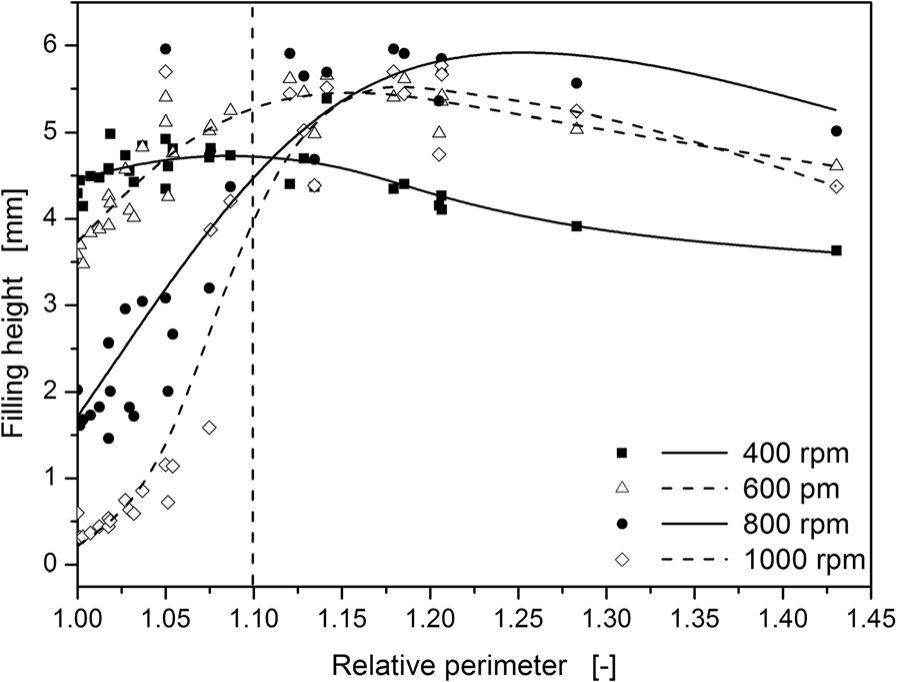
**Filling height as a function of the relative perimeter.** The liquid height is measured vertically at the center of the well bottom with the modified BioLector measurement system at a total liquid filling volume of 500 μL. The dashed line represents the transition from in-phase to out-of-phase liquid behavior. The perimeters of the different well geometries are related to the perimeter of the round geometry with the same well cross-section area. Shaking diameter: 3 mm, temperature: 25°C, shaking frequency: 400 – 1000 rpm.

In Figure 4 the measured liquid heights in microtiter plates are shown for an exemplary filling volume of 500 μL and different relative perimeters and shaking frequencies. For the unbaffled round geometry (relative perimeter = 1.0) the liquid height decreases with increasing shaking frequency (follow data points on the y-axis top down in Figure 4). These measurements agree with the theory that the liquid rises up the well wall the higher the centrifugal forces are and, simultaneously, drains off the well bottom. However, for all elevated perimeters < 1.1, the liquid height at the center of the wells is higher compared to the unbaffled geometry at the same shaking frequencies. In this case, more liquid is located on the well bottom due to the higher degree of baffling and the breakup and restricted movement of the bulk liquid. Although the liquid rotation is reduced, the OTR_max_ still increases at these operating conditions (refer to Figure 3) since the breakup of the bulk liquid also increases its surface area and introduces turbulence into the bulk liquid. For relative perimeters higher than 1.1, the liquid level remains more or less constant at high level. An increased degree of baffling completely prevents the rotation of the liquid. The rotating bulk liquid does not exist anymore and nearly all liquid swashes at the center of the well as illustrated by pictures for shake flasks by Büchs [7]. As a consequence, the maximum oxygen transfer capacity is reduced which can be seen for relative perimeters higher than 1.1 in Figure 3. Finally, these results indicate the transition from in-phase to out-of-phase operating condition beyond the dashed lines in Figure 3 and Figure 4. Thus, the existence of optimal well geometries concerning high maximum oxygen transfer capacity in microtiter plates at a relative perimeter of about 1.1 has been confirmed by applying a completely different measurement method (liquid height) compared to the OTR_max_ measurements.

### The relative perimeter as integral parameter to calculate OTR_max_ in microtiter plates

The aforementioned investigations revealed that the OTR_max_ of the investigated microtiter plates increases linearly with the relative perimeter until a maximal value is reached (Figure 3). For relative perimeters larger than 1.1, a constant or even decreasing trend was noticed. This observation is explained above by prevailing out-of-phase conditions. Therefore, a correlation to calculate OTR_max_ values depending on the relative perimeter, shaking frequency and filling volume can only be derived for relative perimeters smaller than 1.1, where regular in-phase conditions exist. By fitting the experimental results with a least square method, the following equation can be obtained:

(2) $$\mathrm{OT}R_{\max}\;=2.5\;\cdot \;10^{-7}\cdot \;\mathrm{Per}i^{6.0}\;\cdot \;n^{2.37}\;\cdot \;V_{L}^{-0.64}\;+\;3.3\;\cdot \;10^{-4}.$$

Equation (2) represents a novel equation to pre-calculate the maximum oxygen transfer capacity OTR_max_ in baffled 48-well microtiter plates at in-phase operating condition. The OTR_max_ is calculated dependent on the relative perimeter of the well geometry (Peri), the shaking frequency (n) and the filling volume (V_L_). From literature it is known that the shaking diameter also has a strong influence on the oxygen transfer in shaken bioreactors [14]. However, for optical on-line measurements in shaken microtiter plates using the BioLector technique, a constant shaking diameter of 3 mm is established [18]. Therefore, the shaking diameter in this study was kept constant to 3 mm and the influence of the shaking diameter is not considered in Eq. (2). The constant second term in Eq. (2) is explained by the fact that a basal oxygen transfer is accomplished by sole diffusion at static condition (without any convective flow). This effect has originally been discovered by Hermann et al. [13] for 96-well plates and later been verified by Kensy et al. for 48-well plates [17].

**Figure 5:**
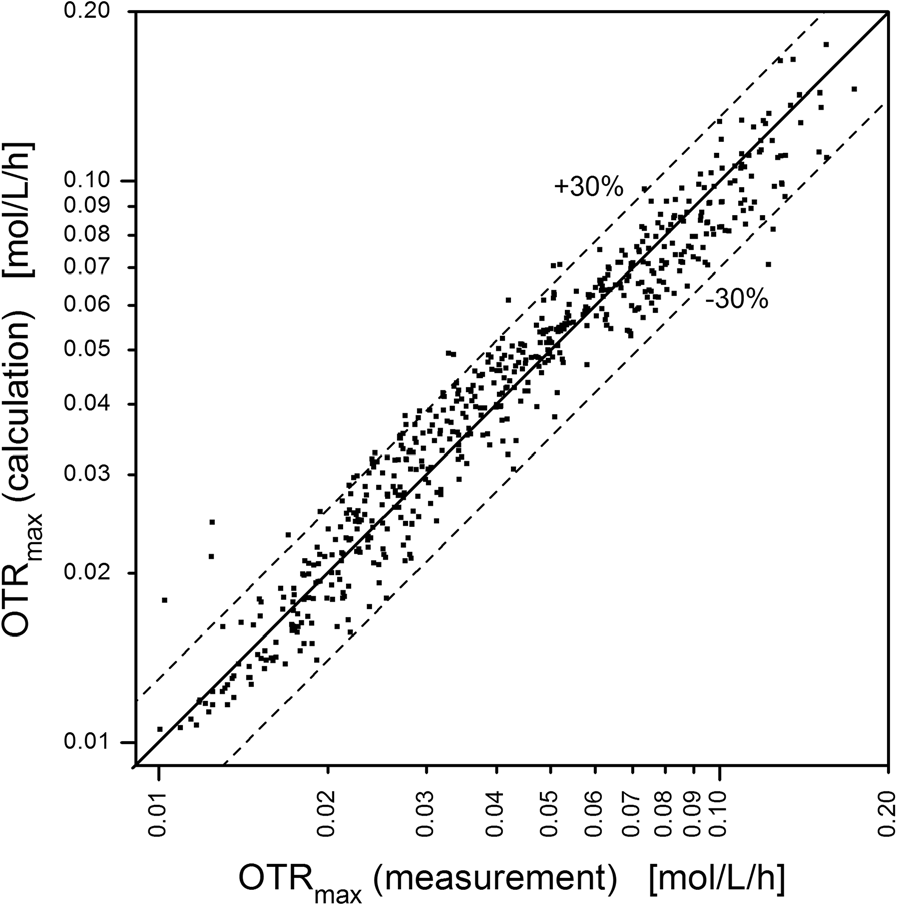
**Comparison of calculated and measured OTR**_**max **_**values.** Only well geometries with a relative perimeter of < 1.1 are considered. The OTR_max_ values are calculated according Eq. (2). Dashed lines indicate a standard deviation of ± 30%.

With Eq. (2) the OTR_max_ values of the different geometries in Figure 1 were calculated and plotted over the values obtained by the oxygen transfer measurements. Figure 5 illustrates the comparison of the measured and calculated OTR_max_ values. It can be seen that all measured data for in-phase operating condition fit to the calculated data within an accuracy of ± 30%. The few outliers which can be seen in Figure 5 do not follow a systematic behavior and cannot be assigned to certain well geometries or operating conditions. Consequently, these outliers are caused by measurement errors in the determination of the OTR_max_ based on optical measurements. Such a correlation can only be derived from a high quantity of experimental data. To reduce the experimental effort, we just considered 48-well microtiter plates in this study. Experimental data from shake flasks are much more time consuming to obtain and less reproducible due to the variability of the baffles, as explained in the introduction. Furthermore, an experimental survey of our results in microtiter plate formats, which differ from the 48-well dimensions, is a topic for further investigations. Thereby, the height of the well geometry will not have a direct influence on the oxygen transfer. It just limits the maximum liquid height in the well and, thus, the maximum shaking frequency. The only defining parameter for different well geometries is already incorporated in our correlation, since the perimeter of the baffled well geometry has been defined as its relative value compared to the perimeter of the round geometry with the same well area (Eq. (1)). By defining the perimeter in that relative way, the obtained results, i.e. the calculation of the OTR_max_ up to a relative perimeter of 1.1, can be transferred to larger well geometries. However, due to changing surface-to-volume ratios, an adaptation of Eq. (2) can be expected, if other microtiter plate formats are used. To complete the discussion, it has to be emphasized that Eq. (2) is restricted so far to a shaking diameter of 3 mm and liquids of water-like viscosities. Furthermore, the influence of surface tension, which is observed in small volume microtiter plates (e.g. 96-well plates), is not taken into account just as the influence of the material surface properties is not considered, which might influence the oxygen transfer as observed in shake flasks [14]. However, from current state of knowledge it is legitimate to assume that the relative perimeter can also be used as key parameter to describe different well formats. Keeping in mind that the investigated baffled geometries vary significantly in number, size and shape, Eq. (2) represents a substantial improvement to characterize the oxygen transfer capacity in small scale shaken bioreactor systems. It is the first time that the maximum oxygen transfer capacities in shaken baffled bioreactors were quantitatively described. With the experience made with 48-well microtiter plates in future work also the effect of baffles in shakes flasks made of glass or plastic material will be investigated.

## Conclusion and outlook

In this work the influence of baffling on the maximum oxygen transfer capacity in 48-well microtiter plates has quantitatively been investigated. The relative perimeter of the cross-section area was chosen as geometric key parameter to correlate the maximum oxygen transfer capacity with the shaking frequency and filling volume. In agreement with the qualitative results of Funke et al [12], an optimum maximum oxygen transfer capacity could be found at a relative perimeter of 1.1. Furthermore, optical measurements of the liquid height at the center of the wells were conducted to obtain additional information about the prevailing operating condition. It could be shown that undesired out-of-phase conditions exist if the relative perimeter exceeds values of 1.1. With this investigation, a phenomenological explanation for the measured mass transfer values could be given. Finally, an empirical correlation was derived to calculate the maximum oxygen transfer capacity in baffled microtiter plates depending on the shaking frequency and filling volume. The relative perimeter as geometric key parameter includes the specific properties of the baffles. Therefore, the degree of baffling in microtiter plates can now quantitatively be described for new geometries if liquids with water-like viscosities are used and effects caused by surface tension are negligible. The obtained correlation is limited to microtiter plates shaken at a shaking diameter of 3 mm. Further investigations in other shaken bioreactors like shake flasks or large shaken barrels have to be carried out to extend the correlation to a more universal equation. The influence of specific effects in small scale bioreactors, e.g. caused by surface tension, could also be investigated in future studies.

## Materials and methods

### Baffled well geometries

A total of thirty different well geometries, varying in number, size and shape of the baffles, were investigated in this work. The baffled geometries shown in Figure 1 have been realized in the wells of 48-well microtiter plates by introducing rectangular or rounded wall structures. The dimensions of the wells were chosen as such that the cross sectional area is always equivalent (112 mm^2^) to the round reference geometry.

Prototypes of microtiter plate bodies were fabricated out of a 20 mm thick acrylic glass plate (polymethyl methacrylat, PMMA) with outer dimensions of 128 mm × 85 mm by laser cutting. To seal the bottom, a PMMA plate of 2 mm thickness was glued onto the bottom of the prototype bodies. As illustrated in Figure 1A, 3 different sets of well geometries represent a gradual transition from the most pronounced baffling (square and pentagon geometry, respectively) to the least pronounced baffling (round geometry). The transition was realized in 3 different ways. First, starting at square geometry, the number of edges is continuously increased. Second and third, starting at square and pentagon geometry, the edges are more and more rounded. Moreover, Figure 1B shows 3 groups of well designs attained by introducing different types of baffles in round or edges well geometries. For further details regarding the investigated well geometries refer to [12].

### Optical measurement system

The BioLector technique was used to determine the liquid height at the well center of the microtiter plate as well as the maximum oxygen transfer capacity OTR_max_ of the different baffled geometries. This optical measurement system enables non-invasive fluorescence and scattered light measurements in shaken microtiter plates [18]. The shaking process of the microtiter plate is not interrupted and, thus, disturbing influences during measurement are avoided. Thereby, a quasi-continuous measurement in shaken microtiter plates is realized. In this study, a slightly modified BioLector system was used which consists of an orbital shaker (based on Lab-Shaker LS-W, Kühner AG, Basel, Switzerland), a x-y linear drive (Bosch Rexroth AG, Lohr am Main, Germany), a custom-made filter fluorescence spectrometer (PreSens GmbH, Regensburg, Germany) and a computer. The orbital shaker was modified to realize a shaking diameter of 3 mm and shaking frequencies of up to 1000 rpm. A hood was placed above the microtiter plate on the shaker tray. The hood was continuously flushed with humidified air to reduce evaporation. To avoid the influence of a cover foil, no additional cover except the hood was used. For further information regarding the optical measurement device refer to [12].

### Characterization of oxygen transfer with a 0.5 M sulfite system

The maximum oxygen transfer capacity OTR_max_ of the different well geometries was characterized by applying a slightly modified sulfite oxidation method originally developed by Hermann et al. [13,17,19]. As described in detail by Funke et al. [12], the pH-indicator of the original recipe [19] was replaced by a 2 · 10^−8^ M hydroxypyrenetrisulfonic acid (HPTS) solution (Fluka, Buchs, Switzerland), which shows pH dependent fluorescence properties. Corresponding to the mentioned sulfite oxidation method, the time t_ox_ is measured until the oxidation of the 0.5 M sodium sulfite to sulfate is completed. At the end of the reaction the pH value in the test solution drops sharply. The fluorescence of HPTS, excited at 420 nm and measured above 515 nm, decreases with decreasing pH. Thus, the pH drop and, therefore, the reaction time can be exactly monitored by means of optical measurements. For known reaction time (t_ox_), initial concentration of sulfite (c_sulfite_) and stoichiometric coefficient for oxygen (ν_O2_ = 0.5), the oxygen transfer rate (OTR) from gas to liquid phase can be calculated as follows:

(3) $$\mathrm{OTR}\;=\;k_{L}a\;\cdot \;\left(c_{O_{2}}^{*}-\;c_{L}\right)=\;c_{\mathrm{sulfite}}\;\cdot \;\nu_{O_{2}}/t_{\mathrm{ox}}.$$

In Eq. (3) the volumetric mass transfer coefficient k_L_a as well as the oxygen concentrations in the bulk liquid (c_L_) and the oxygen concentration at the liquid gas interface (c_O2_^*^) are unknown. According to Weisenberger and Schumpe [20], the oxygen concentration at the liquid gas interface c_O2_^*^ can be calculated as the product of oxygen solubility in the liquid and the oxygen partial pressure in the gas phase (L_O2_ = 8.35 · 10^−4^ mol∙L^−1^∙bar^−1^ at 25°C, p_G_ = 0.2095 bar). Furthermore, Hermann et al. [13] determined the reaction constant k_1_ for the first-order kinetic for the sulfite oxidation out of experiments in stirred tank reactors to k_1_ = 2.385 h^−1^. By using a mass balance for oxygen transfer into the bulk liquid of a shaken microtiter plate, the oxygen concentration in the bulk liquid c_L_ can be determined if the OTR is known [13]:

(4) $$c_{L}=\;\mathrm{OTR}/k_{1}.$$

Combining Eq. (3) and Eq. (4), the volumetric mass transfer coefficient k_L_a can be calculated as follows:

(5) $$k_{L}a\;=\;\mathrm{OTR}/\left(L_{O_{2}}\;\cdot \;p_{G}-\;\mathrm{OTR}/k_{1}\right).$$

If the oxygen concentration in the bulk liquid is close to zero, the OTR becomes maximal. Consequently, the maximum oxygen transfer capacity OTR_max_ can be calculated by the following equation:

(6) $$\begin{array}{l} \mathrm{OT}R_{\max}\;=k_{L}a\;\cdot \;c_{O_{2}}^{*}\\ \;=\;\mathrm{OTR}\;\cdot \;L_{O_{2}}\;\cdot \;p_{G}/\left(L_{O_{2}}\;\cdot \;p_{G}\;-\;\mathrm{OTR}/k_{1}\right). \end{array}$$

Using Eq. (6), the maximum oxygen transfer capacity can now be obtained indirectly through measurement of the oxygen transfer rate.

### Detection of out-of-phase conditions through liquid height measurements

The liquid height at the well center of the microtiter plate was determined by measuring the fluorescence intensity of a 1.25 · 10^−6^ M fluorescein solution (sodium salt, Fluka, Buchs, Switzerland) in 0.2 M sodium phosphate buffer (pH 7) (Roth, Karlsruhe, Germany). The fiber optics of the BioLector prototype was vertically installed underneath the wells. All experiments to determine the liquid height were conducted at a constant filling volume of V_L_ = 500 μL and different shaking frequencies of 400 – 1000 rpm. The fluorescence was excited at 420 nm (bandpassfilter ± 10 nm) and detected above 515 nm (cut-of filter). For calibration, different filling volumes of the fluorescein solution were filled into the microtiter plate prototypes. The fluorescence signal was measured at a shaking frequency of 200 rpm, where no liquid movement occurs [13]. Since the cross-section area for each geometry is constant (112 mm^2^), the liquid height can be calculated depending on the filling volume. The fluorescence signal of the fluorescein solution decreases with decreasing liquid height. Thus, a geometry-independent correlation of fluorescence signal and liquid height at the well center is obtained.

### Fitting of measurement data by applying the L-curve criterion

For the graphical presentation both the experimental data of the maximum oxygen transfer capacity and of the filling height at the well center were fitted by using smoothing splines. The regularization parameter α of the smoothing spline fit was determined by using the L-curve criterion [21,22]. The L-curve is a plot of the norm of the regularized solution (S) representing the smoothness of the curve (Eq. (7)) versus the norm of the corresponding residual (R) representing the data error (Eq. (8)). A good compromise between both norms, i.e. a suitable regularization parameter can be found at the “corner” of the L-curve plot. This is the point where the curve is closest to the origin.

(7) $$\begin{array}{l} S=\left|\left|\vec{X}\right|\right|\\ \;=\;\sqrt{{\left(X_{\mathrm{spline},1}\right)}^{2}+\;{\left(X_{\mathrm{spline},2}\right)}^{2}+\ldots +\;{\left(X_{\mathrm{spline},i}\right)}^{2}} \end{array}$$

(8) $$\begin{array}{l} R=\left|\left|\vec{X}\right|\right|\\ \;=\;\sqrt{{\left(X_{\mathrm{spline},1}-\;X_{1}\right)}^{2}+\;{\left(X_{\mathrm{spline},2}-\;X_{2}\right)}^{2}+\ldots +\;{\left(X_{\mathrm{spline},i}-\;X_{i}\right)}^{2}} \end{array}$$

A slight modification compared to the literature was used when applying the L-curve criterion. The square of the norm of the corresponding residual (R^2^) was doubled in order to obtain a fitting better representing the experimental data. Thus, the suitable regularization parameter α was defined as the radius of a circle around the origin which touches the L-curve at the closest point to the origin. This led to the following minimization problem:

(9) $$\mathrm{mi}n_{\alpha }\left|\left|2\cdot \;R_{\alpha }^{2}+\;S_{\alpha }^{2}\right|\right|.$$

Equations (7) to (9) were implemented into an algorithm and solved by applying the MATLAB software package (Version 7.8.0; The Mathworks, MA, USA).

## Abbreviations

HPTS: 8-Hydroxypyrene-1,3,6-trisulfonic acid; MTP: Microtiter plate; α: Regularization parameter [-]; c_L_: Oxygen concentration in the liquid [mol/L]; c_O2_^*^: Oxygen concentration at the gas-liquid interface [mol/L]; c_sulfite_: Sulfite concentration [g/L]; k_1_: First-order kinetic reaction constant [1/h]; k_L_a: Volumetric mass transfer coefficient [1/h]; L_O2_: Oxygen solubility in the liquid [mol/L]; n: Shaking frequency [1/min]; OTR: Oxygen transfer rate [mol/L/h]; OTR_max_: Maximum oxygen transfer capacity [mol/L/h]; p_G_: Oxygen partial pressure in the gas phase [bar]; Peri: Normalized perimeter of the baffled geometry [-]; Perimeter_baffled_: Perimeter of the baffled geometry [mm]; Perimeter_round_: Perimeter of the round reference geometry [mm]; Ph: Phase-Number [-]; R: Residual, representing the data error [-]; S: Regularized solution, representing the smoothness of the curve [-]; t_ox_: Reaction time [s]; V_L_: Filling volume [μL]; ν_O2_: Stoichiometric coefficient of oxygen [-]; X_spline,i_: Data point of the spline [-], in this study substituted by calculated data of OTR_max_; X_i_: Data point of measurement data [-], in this study substituted by measured data of OTR_max_.

## Competing interests

The authors declare that they have no competing interests.

## Authors’ contributions

CL is the author of the final version of this study. MF is responsible for the experimental results. He also drafted the first version of the manuscript. CL, MF and JB masterminded the study and participated in its design, coordination, and drafting and finalizing of the manuscript. SH kindly assisted in fitting the experimental data. SD supported in conducting and evaluating experimental results. All authors read and approved the final manuscript.
